# Intraoperative mechanical power and postoperative pulmonary complications in low-risk surgical patients: a prospective observational cohort study

**DOI:** 10.1186/s12871-024-02449-1

**Published:** 2024-02-27

**Authors:** Mohamad El-Khatib, Carine Zeeni, Fadia M. Shebbo, Cynthia Karam, Bilal Safi, Aline Toukhtarian, Nancy Abou Nafeh, Samar Mkhayel, Carol Abi Shadid, Sana Chalhoub, Jean Beresian

**Affiliations:** 1 Department of Anesthesiology and Pain Medicine, American University of Beirut Medical Center, PO-Box: 11-0236, Beirut, 1107 2020 Lebanon; 2 Department of Emergency Medicine, American University of Beirut Medical Center, Beirut, Lebanon

**Keywords:** General anesthesia, Mechanical power, Mechanical ventilation, Perioperative care, Postoperative pulmonary complications

## Abstract

**Background:**

Inadequate intraoperative mechanical ventilation (MV) can lead to ventilator-induced lung injury and increased risk for postoperative pulmonary complications (PPCs). Mechanical power (MP) was shown to be a valuable indicator for MV outcomes in critical care patients. The aim of this study is to assess the association between intraoperative MP in low-risk surgical patients undergoing general anesthesia and PPCs.

**Methods:**

Two-hundred eighteen low-risk surgical patients undergoing general anesthesia for elective surgery were included in the study. Intraoperative mechanical ventilatory support parameters were collected for all patients. Postoperatively, patients were followed throughout their hospital stay and up to seven days post discharge for the occurrence of any PPCs.

**Results:**

Out of 218 patients, 35% exhibited PPCs. The average body mass index, tidal volume per ideal body weight, peak inspiratory pressure, and MP were significantly higher in the patients with PPCs than in the patients without PPCs (30.3 ± 8.1 kg/m^2^ vs. 26.8 ± 4.9 kg.m^2^, *p* < 0.001; 9.1 ± 1.9 ml/kg vs. 8.6 ± 1.4 ml/kg, *p* = 0.02; 20 ± 4.9 cmH_2_O vs. 18 ± 3.7 cmH_2_O, *p* = 0.001; 12.9 ± 4.5 J/min vs. 11.1 ± 3.7 J/min, *p* = 0.002). A multivariable regression analysis revealed MP as the sole significant predictor for the risk of postoperative pulmonary complications [OR 1.1 (95% CI 1.0–1.2, *p* = 0.036].

**Conclusions:**

High intraoperative mechanical power is a risk factor for developing postoperative pulmonary complications. Furthermore, intraoperative mechanical power is superior to other traditional mechanical ventilation variables in identifying surgical patients who are at risk for developing postoperative pulmonary complications.

**Clinical trial registration:**

NCT03551899; 24/02/2017.

## Introduction

The incidence of postoperative pulmonary complications (PPCs) in noncardiac surgical patients undergoing abdominal, orthopedic, and neurological procedures under general anesthesia ranges between 9.7% and 34% [1, 2]. These PPCs can lead to increased early postoperative morbidity, mortality, intensive care unit (ICU) admission, and ICU/hospital length of stay [1, 2]. Inappropriate settings of intraoperative mechanical ventilation (MV) variables have been implicated in the incidence of PPCs in patients with both healthy and diseased lungs [3, 4]. Intraoperative tidal volume (V_T_), positive end-expiratory pressure (PEEP), peak inspiratory (PIP) and peak alveolar (P_plateau_) pressures as well as driving pressures (ΔP, the difference between P_plateau_ and PEEP) are key variables of intraoperative settings of MV. When not optimized intraoperatively, these variables can predispose patients to ventilator-induced lung injury (VILI) and subsequently increase the risk for PPCs [5–7].

In 2016, the concept of mechanical power (MP) was introduced for the first time as an essential cause of injury during invasive MV in patients with acute respiratory distress syndrome (ARDS) [8]. It was reported that MP, which reflects the energy dissipated by the mechanical ventilator onto the respiratory system over time and subsequently the intensity of MV, may help in estimating the contribution of the different ventilator-related variables (i.e., V_T_, RR, PEEP, and P_plateau_) of lung injury [8]. Furthermore, when compared to other classical variables of mechanical ventilation, such as V_T_, PEEP, P_plateau_, and ΔP, MP was shown to be a superior predictor of outcomes from MV, and high MPs are associated with high morbidity and mortality in ICU patients with ARDS [8].

Recently, the first study evaluating the association between MP and PPCs in patients undergoing general anesthesia for noncardiothoracic and nonintracranial surgeries showed that exposure to high MPs was independently associated with increased risks of PPCs and acute respiratory failure [9]. The study, which was a post hoc analysis of a large randomized clinical trial [10], combined both high- and low-risk surgical patients older than 40 years old who were ventilated with volume-controlled ventilation using only two specific tidal volumes of either 6 mL/kg or 10 mL/kg of ideal body weight, a fixed PEEP of 5 cmH2O, and used PIP instead of P_plateau_ for the determination of MP [9]. Another recent retrospective cohort study showed that higher intraoperative MP was statistically associated with a greater risk of postoperative respiratory failure requiring reintubation [11]. Again, the study did not stratify patients as per their preoperative risks for developing PPCs and did not use P_plateau_ exclusively for the determination of MP in all patients but used PIP as a surrogate of P_plateau_ whenever P_plateau_ values were not available [11]. Including a mix of patients with high- and low-risks for developing PPCs as well as using PIP as a surrogate of P_plateau_ in the determination of MP can negatively impact the accuracy of MP and ultimately some of the conclusions reported thus far [8, 12]. The aim of the current study was to assess the association between “accurate” intraoperative MP that relies exclusively on P_plateau_ values and the development of PPCs in adult low-risk surgical patients receiving general anesthesia and MV for major noncardiac surgeries.

## Methods

This prospective observational study was conducted between January 2017 and July 2021 at the American University of Beirut–Medical Center. The study was approved by the Institutional Review Board and registered in Clinicaltrilas.gov (NCT03551899; 24/02/2017). Written informed consent was obtained from all patients who participated in the study.

Patients with ASA I and II, above 18 years old, scheduled to undergo noncardiac surgeries under general anesthesia and invasive MV for at least 2 h, and who are at low risk for developing postoperative pulmonary complications (i.e.; Assess Respiratory Risk in Surgical Patients in Catalonia (ARISCAT) score < 26 [13, 14]) were considered for inclusion in the study. Patients were excluded if they had obstructive sleep apnea or were pregnant. Patients on whom P_plateau_ was missing or could not be obtained were also excluded.

Upon arrival to the induction room, intravenous access was secured for all patients and standard ASA monitoring, including electrocardiogram and heart rate, noninvasive blood pressure, respiratory rate and oxygen saturation, was applied and maintained throughout the surgery.

All patients received a standard general anesthesia protocol consisting of preoxygenation followed by 2 mg/kg propofol, 2 µg/kg fentanyl, and 0.6 mg/kg rocuronium. After tracheal intubation, anesthesia was maintained by remifentanil 0.05–0.2 µg/kg/min and sevoflurane with or without nitrous oxide to achieve a minimum alveolar concentration (MAC) of at least 1. Anesthesia and mechanical ventilation were provided using the GE Avance CS^2^ Anesthesia Delivery System (GE Healthcare, USA). The research team did not interfere with the selection of mode of ventilation and specific mechanical ventilation settings which were left at the discretion of the anesthesiologist in charge of the case who were free to make any changes in mechanical ventilation settings with the endpoints of maintaining oxygen saturation of at least 95% and an end-tidal CO_2_ between 30–40 mmHg as per our anesthesia guidelines.

At the beginning of the surgery, and every 30 min thereafter, a member of the research team collected all relevant mechanical ventilation data, which included mode of ventilation, V_T_, PEEP, PIP, respiratory rate (RR), inspiratory to expiratory time ratio (I:E), and P_plateau_. The P_plateau_ was determined by applying an end-inspiratory occlusion of at least 0.5 s to achieve a stable plateau in the airway pressure waveform [15]. The highest values for each ventilatory parameter were used for data analysis. ΔP was determined (ΔP = P_plateau_-PEEP) and MP was calculated as previously established for volume control (MP = 0.098 × RR x V_T_ x [PIP – 0.5 x (P_plateau_ – PEEP)]) and pressure control ventilation (MP = 0.098 × RR x V_T_ x [PEEP + pressure above PEEP]) [16–18]. Respiratory system dynamic compliance (Cdyn) normalized to body mass index was calculated as (V_T_/(PIP-PEEP))/BMI while respiratory system static compliance (Cstat) normalized to body mass index was calculated as (V_T_/(P_plateau_-PEEP))/BMI. All recorded and derived data were not shared with the anesthesia team clinically in charge of the case. Other relevant data collected included patient demographic and baseline physiological parameters.

At the end of the surgery, patients were extubated according to our institutional protocol that necessitates patients to be normothermic, physiologically stable, awake, cooperative, spontaneously breathing, and with no residual curarization (i.e., train of four ˃95%). Following extubation, patients were transferred to the post anesthesia care unit (PACU) for observation. The research team maintained a close follow-up on all patients upon their arrival to the PACU, throughout their hospital stay, and up to 7 days post discharge, to assess for the occurrence of any PPCs. PPCs which were identified by the medical team in charge of the patients included unplanned oxygen supplementation (need for oxygen administration for more than 1 day due to ≥ 5% absolute drop from preoperative SpO_2_ value), atelectasis and/or pulmonary congestion (as per postoperative compared to preoperative X-ray), pneumonia (presence of new or progressive radiographic infiltrate and at least two of the following three clinical features: 1. fever > 38 °C; 2. leucocytosis or leukopenia; 3. purulent secretions), ARDS (as per the Berlin definition), and need for invasive and/or noninvasive MV. The occurrence of at least one of the latter specified list throughout the whole follow-up period was considered a PPC event.

All collected variables were tested for normality using Kolmogorov–Smirnov-Lilliefors. Preoperative data (age, weight, height, sex, ASA physical status, smoking status, preoperative SpO_2_, ARISCAT score, presence of anemia, history of pulmonary or cardiovascular diseases, any other chronic morbidity, and the presence of respiratory infection during the month preceding surgery) were collected preoperatively and presented either as the mean ± SD, median [range], or frequency (percentage). Continuous data collected intraoperatively including, V_T_, C_dyn_/BMI, C_stat_/BMI, PEEP, PIP, P_plateau_, ΔP, fraction of inspired oxygen, RR, and MP, are presented as either medians with ranges or means ± standard deviations.

Proportions were compared using Chi-squared or Fisher exact tests and continuous data were compared using the t-test. Simple regression analysis was performed to identify intraoperative predictors of PPCs with p-value less than 0.2; these variables were included in the final multivariable regression analysis model. Effects were expressed as an average odds ratio (OR) with 95% confidence interval. All analyses were performed using SPSS software. Statistical significance was considered at *p* < 0.05.

## Results

Two-hundred eighteen patients were enrolled in the study. Demographics, physiological variables, surgical data and intraoperative MV parameters for all patients and patients with and without PPCs are presented in Table 1. Seventy-seven (35%) patients exhibited at least one PPC event. The types of PPCs included the need for unplanned oxygen supplementation for more than 1 day via a nasal cannula or facemask (73 patients), the use of high flow nasal cannula oxygen therapy (2 patients), the use of noninvasive ventilatory support (1 patient), and the use of invasive mechanical ventilation (1 patient). The average body mass index (BMI), V_T_/IBW, PIP, and MP were significantly lower in patients with no PPCs than in patients with PPCs while the dynamic respiratory system compliance normalized to body mass index (C_dyn_/BMI) was significantly higher in patients with no PPCs than in patients with PPCs (Table 1).

**Table 1:** Demographics and baseline physiological parameters

	All patients	PPC	No PPC	*p*-value
	(*n* = 218)	(*n* = 77)	(*n* = 141)	
Age, yrs	42.6 ± 14.4	42.6 ± 14.4	42.6 ± 14.4	0.11
BMI (kg/m^2^)	28.0 ± 6.4	30.3 ± 8.1	26.8 ± 4.9	< 0.001
Gender				
M, n (%)	107 (49%)	41 (53%)	68 (47%)	0.4
F, n (%)	111 (51%)	36 (47%)	78 (53%)	
ASA				
I, n (%)	50 (23%)	12 (16%)	38 (27%)	0.07
II, n (%)	168 (77%)	65 (84%)	103 (73%)	
SpO_2_	99.0 ± 1.1	99.0 ± 1.2	99.2 ± 1.0	0.07
Surgery type				
Orthopedic	73	31 (40%)	42 (30%)	
ENT	34	10 (13%)	24 (17%)	
Breast	23	7 (9%)	16 (11%)	0.60
General (non-abdominal)	23	7 (9%)	16 (11%)	
Laparoscopic abdominal	17	7 (9%)	10 (7%)	
Others	48	15 (20%)	33 (24%)	
ARISCAT Score	10.0 ± 8.2	11.1 ± 8.0	9.5 ± 8.4	0.18
C_dyn_/BMI (ml/cmH_2_O)/(kg/m^2^)	1.44 ± 0.60	1.27 ± 0.49	1.52 ± 0.63	0.003
C_stat_/BMI (ml/cmH_2_O)/(kg/m^2^)	1.63 [0.2–7.0]	1.5 [0.2–7.0]	1.7 [0.5–5.3]	0.047
V_T_/IBW (ml/kg)	8.8 ± 1.6	9.1 ± 1.9	8.6 ± 1.4	0.02
RR (breaths/min)	12.0 ± 1.7	12.1 ± 1.6	12.0 ± 1.7	0.51
PEEP (cmH_2_O)	5 [0–5]	5 [0–5]	5 [0–5]	0.63
PIP (cmH_2_O)	18.7 ± 4.3	20.0 ± 4.9	18.3 ± 3.7	0.001
P_plateau_ (cmH_2_O)	16.5 ± 4.8	17.4 ± 5.5	16.1 ± 4.3	0.06
ΔP (cmH_2_O)	13.0 ± 4.7	13.8 ± 5.2	12.6 ± 4.4	0.09
MP (J/min)	11.8 ± 4.1	12.9 ± 4.5	11.1 ± 3.7	0.002

Data is presented as either mean ± SD, median [range], or number (percentage)

*PPC* Postoperative pulmonary complication, *C*_*dyn*_*/BMI* Dynamic compliance/body mass index, *C*_*stat*_*/BMI* Static compliance/body mass index, *V*_*T*_*/IBW* Tidal volume/ideal body weight, *RR* Respiratory rate, *PEEP* Positive end-expiratory pressure, *PIP* Peak inspiratory pressure, *P*_*plateau*_ Plateau pressure, *ΔP* Driving Pressure, *MP* Mechanical power

**Fig. 1:**
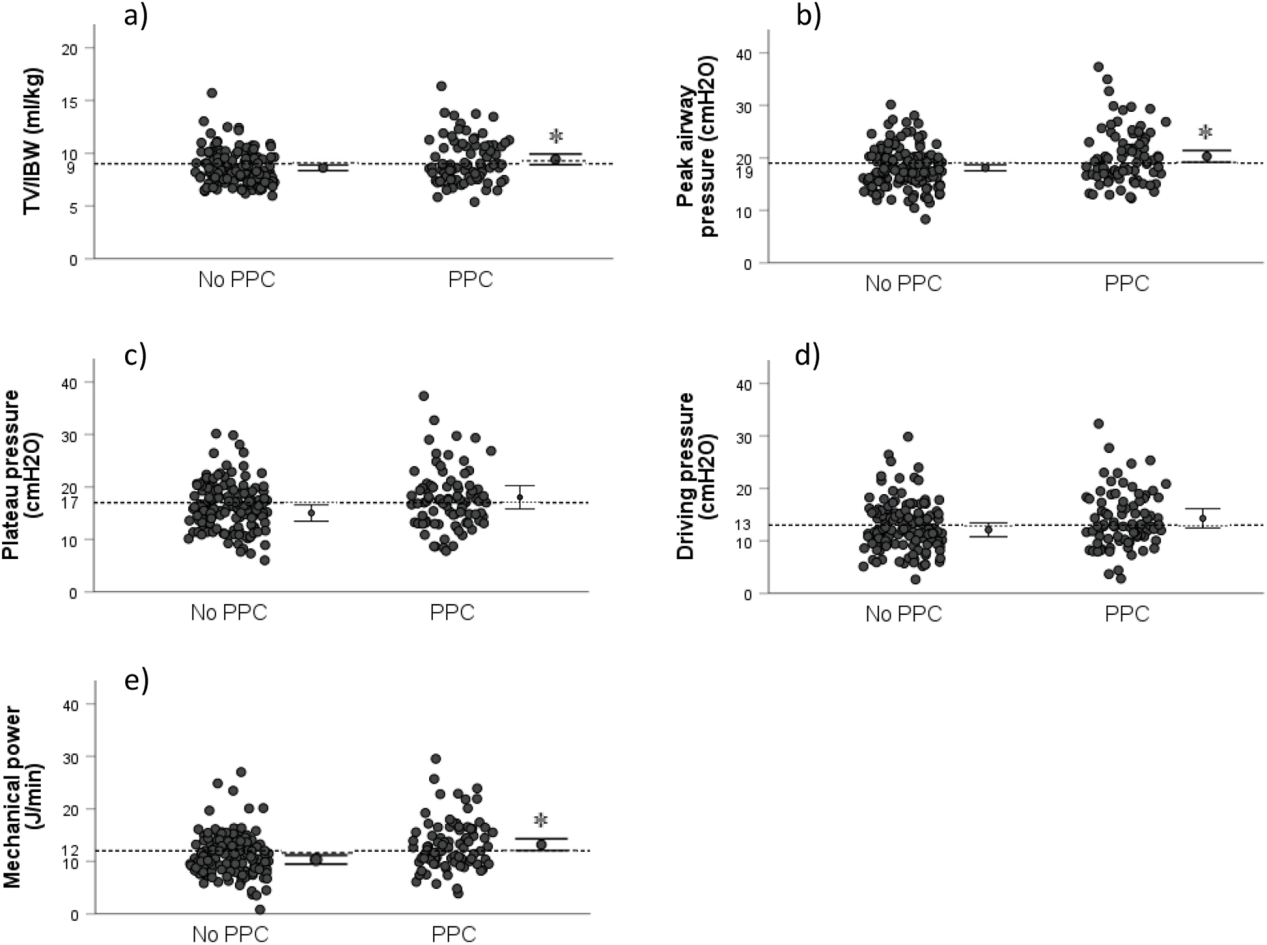
Scattergrams for (**a**) tidal volume per ideal body weight (V_T_/IBW); **b** peak airway pressure (PIP); **c** plateau pressure (P_plateau_); **d** driving pressure (ΔP); **e** mechanical power (MP) in patients with and without postoperative pulmonary complications (PPCs)

The distributions of V_T_/IBW, PIP, P_plateau_, ΔP, and MP in patients with and without PPCs are shown in Fig. 1. The cutoffs with the highest sensitivity for separating the mean values between patients with and without PPCs were 9 ml/kg for V_T_/IBW, 19 cmH_2_O for PIP, 17 cmH_2_O for P_plateau_, 13 cmH_2_O for ΔP, and 12 J/min for MP.

The odds ratios (ORs) of MV variables for the risk of developing PPCs are presented in Table 2. MP and P_plateau_ had the highest and only statistically significant odds ratios for risks of developing PPCs (Table 2). Simple logistic regression analysis that included V_T_/IBW, P_plateau_ and MP (since MP and PIP as well as P_plateau_ and ΔP had high collinearity) indicated both V_T_/IBW and MP to be significant independent indicators of PPCs. However, multivariable logistic regression revealed MP as the only significant intraoperative predictor for developing PPCs [OR 1.1 (95% CI: 1.0–1.2, *p* = 0.036] (Table 3).

**Table 2:** Intraoperative mechanical ventilation settings in the patients who exhibited postoperative pulmonary complications

	All patients	PPC	Odds ratio	*p*-value
	(*n* = 218)	(*n* = 77)	[95% CI]	
V_T_/IBW (ml/kg)			1.6 [0.9–2.8]	0.11
< 9 ml/kg	135	42 (31.1%)		
≥ 9 ml/kg	83	35 (42.2%)		
RR (breaths/min)			0.9 [0.5–1.6]	0.65
< 12 breaths/min	61	23 (37.7%)		
≥ 12 breaths/min	157	54 (34.4%)		
PEEP (cmH_2_O)			1.1 [0.6–2.0]	0.77
< 5 cmH_2_O	74	25 (33.8%)		
≥ 5 cmH_2_O	144	52 (36.1%)		
PIP (cmH_2_O)			1.7 [0.9–2.9]	0.09
< 19 cmH_2_O	125	38 (30.4%)		
≥ 19 cmH_2_O	93	39 (41.9%)		
P_plateau_ (cmH_2_O)			2.1 [1.0–4.0]	0.04
< 17 cmH_2_O	173	55 (31.6%)		
≥ 17 cmH_2_O	45	41 (48.9%)		
ΔP (cmH_2_O)			1.4 [0.8–2.5]	0.26
< 13 cmH_2_O	114	36 (31.6%)		
≥ 13 cmH_2_O	104	41 (39.4%)		
MP (J/min)			1.8 [1.0–3.2]	0.04
< 12 J/min	128	38 (29.7%)	38 (29.7%)	
≥ 12 J/min	90	39 (43.3%)	39 (43.3%)	

Data is presented as frequency (%) of corresponding “All patients”

*PPC* Postoperative pulmonary complication, *V*_*T*_*/IBW* Tidal volume/ideal body weight, *RR* Respiratory rate, *PEEP* Positive end-expiratory pressure, *PIP* Peak inspiratory pressure, *P*_*plateau*_ Plateau pressure, *ΔP* Driving pressure, *MP* Mechanical power

**Table 3:** Regression analysis of intraoperative ventilator settings on postoperative pulmonary complications

	No PPC	PPC	Odds ratio	*p*-value
			[95% CI]	
Simple logistic regression				
V_T_/IBW (ml/kg)	8.6 ± 1.4	9.1 ± 1.9	1.2 [1.0–1.5]	0.024
P_plateau_ (cmH_2_O)	16.1 ± 4.3	17.4 ± 5.5	1.1 [0.9–1.1]	0.059
MP (J/min)	11.1 ± 3.7	12.9 ± 4.5	1.1 [1.0–1.2]	0.003
Multiple logistic regression				
V_T_/IBW (ml/kg)	8.6 ± 1.4	9.1 ± 1.9	1.1 [0.9–1.4]	0.187
P_plateau_ (cmH_2_O)	16.1 ± 4.3	17.4 ± 5.5	1.0 [0.9–1.1]	0.982
MP (J/min)	11.1 ± 3.7	12.9 ± 4.5	1.1 [1.0–1.2]	0.036

Data is presented as mean ± standard deviation or odds ratio [confidence interval]

*V*_*T*_*/IBW* Tidal volume/ideal body weight, *P*_*plateau*_ Plateau pressure, *MP* Mechanical power

## Discussion

Our current study shows that a substantial proportion of noncardiac low-risk surgical patients receiving invasive mechanical ventilation in the operating room are at risk of developing PPCs. These patients apparently receive higher mechanical ventilation intensity as reflected by higher MP compared to patients with no PPCs. Furthermore, the current results show that MP may be superior to other traditional mechanical ventilation variables in identifying noncardiac surgical patients who are at risk for developing PPCs.

To our knowledge, this is the first study that uses “accurate” MP in low-risk surgical patients receiving invasive MV in the operating room and assesses its utility for identifying patients at risk of developing PPCs. In the current study, P_plateau_ rather than PIP was used exclusively in the accurate determination of ΔP and “accurate” MP [8, 16–18]. In only two previous and similar studies, Karalapillai et al. [9] used PIP as a surrogate of P_plateau_ for calculating ΔP and MP, while Santer et al. used a mix of PIP and P_plateau_ in determining ΔP and MP [11]. Both of these approaches carry the risk of generating inaccurate MP values [12].

MP has been recognized as a promising indicator of VILI and predictor of outcomes from MV in critically ill patients [8, 19–21], but little is known about its potential association with VILI and the concomitant outcomes of MV in low-risk surgical patients with healthy lungs undergoing general anesthesia. Marini et al. reported that high MPs at low to normal tidal volumes can significantly amplify both the magnitude and velocity of the stretching forces of the tidal breath leading to parenchymal injuries and the release of inflammatory mediators [19].

Recently, Karalapillai et al. showed in a mixed population of low- and high-risk surgical patients that high intraoperative MP was associated with an increased risk of PPCs [9]. Although our current study shows similar findings, there remain major differences between the two studies that are worth mentioning. First, in the current study, only low-risk surgical patients with ASA I and II and ARISCAT scores of < 26 were considered while Karalapillai et al. included both low and high-risk surgical patients and 52% of their patients were ASA III & IV and 64.2% had an ARISCAT score ≥ 26. Second, in our study we included patients older than 18 years, while Karalapillai et al. included only patients older than 40 years. This resulted in a younger patients population in our study (42 years) compared to the Karalapillai et al. study (64 years). Third, Karalapillai et al. used volume-controlled mode of ventilation with only two preset tidal volumes of 6 mL/kg or 10 mL/kg of ideal body weight while in our study patients were ventilated with either volume-control or pressure-controlled ventilation using a wide range of tidal volumes. Fourth and more importantly, Karalapillai et al. used PIP rather than P_plateau_ for the determination of ΔP and MP in contrast to our study, where we exclusively used P_plateau_ in the determination of ΔP and MP, as appropriately indicated by Gattinoni et al. [8]. Finally, in contrast to the Karalapillai et al. study [9] that used PEEP of 5 cmH_2_O in all patients, our current study did not exclude any patient based on his/her PEEP level.

Another recent study by Santer et al. reported that higher MP during intraoperative MV was statistically associated with greater risks of postoperative respiratory failure requiring reintubation [11]. However, in the study by Santer et al. the authors again used PIP as a surrogate of P_plateau_ whenever the latter was not available and ended up using a mix of P_plateau_ and PIP values for determining MP which can significantly overestimate the “accurate” MP [12]. Additionally, the study by Santer et al. did not stratify patients as per their preoperative risks for developing PPCs. Finally, Santer et al. included patients from 2008 to 2018, i.e., 8 years prior to introducing the concept of mechanical power and only 2 years thereafter.

The observed incidence of at least 1 PPC in the current study was 35%, which is considered higher than that previously reported [20, 21] but similar to more recent findings [4]. The differences in PPCs occurrences could be attributed to patient characteristics, surgical procedures, and PPC definitions. Most of the PPCs observed in the current study consisted of using unplanned oxygen supplementation for greater than one day (94.8%) to correct for a ≥ 5% drop in baseline SpO_2_, while the remaining PPCs included the use of HFNC (2.6%), NIV (1.3%) or MV (1.3%). As such, higher intraoperative MPs reflecting higher intraoperative MV intensity may be an important factor contributing to the development of PPCs through the generation of excessive stress and strain on the lung units and leading to the formation of interstitial and alveolar edema with increased ventilation to perfusion mismatch [22–24]. Also, in the presence of atelectasis, a common occurrence during general anesthesia, higher MP and higher mechanical ventilation intensity may cause further increases in alveolar epithelial permeability, lung edema and inflammation by repetitive collapse and reopening of alveolar units at high power delivery, a phenomenon known as atelectrauma [22].

Recent studies have shown that MP greater than 17 J/min is not only a useful indicator of VILI but also a valuable predictor of patient outcomes from MV and is associated with higher ICU mortality, 30-day mortality, ventilator-free days, and shorter ICU and hospital length of stay [23, 24]. In the current study, the average MP in patients who developed PPCs was higher (MP = 12.9 J/min) than in patients who did not develop PPCs (MP = 11.1 J/min). Furthermore, at a threshold of 12 J/min, MP was found to be a significant predictor of PPCs in our low-risk surgical patients. As expected, the average MP values reported in our study were lower than those reported for ARDS patients since our patients were low-risk surgical patients undergoing elective surgery. Nevertheless, our patients were at risk for increased PPCs when MP exceeded 12 J/min similar to what was previously reported in healthy piglets [25] and every additional 1 J/min increase in MP was associated with 10% higher odds for developing PPCs (OR [95% CI] of 1.1 [1.0–1.2]; *p* = 0.036). None of the patients in our study needed ICU admission, and all patients were successfully managed and discharged from the PACU with no mortality.

In our study, BMI was significantly higher (30.3. kg/m^2^ vs. 26.8 kg/m^2^), and respiratory system dynamic compliance normalized to BMI was significantly lower (1.27 (mL/cmH_2_O)/(kg/m^2^) vs. 1.52 (mL/cmH_2_O/(kg/m^2^)) in patients who developed PPCs than in patients who did not develop PPCs. Increased BMI is known to cause substantial detrimental changes to the volumes, capacities, and mechanics of the lungs and chest wall. Increased BMI can induce decreases in forced vital capacity (FVC), forced exhaled volume in 1 s (FEV_1_), increases in total airway, peripheral, and tissue respiratory system resistances and decreases in lung compliance [26–28]. Any decline in lung mechanics, volumes, or capacities resulting from higher BMI will lead to using higher intraoperative mechanical ventilation intensity and MPs to maintain oxygenation, ventilation, and acid–base balance [8] but not necessarily into using higher or stressful intraoperative V_T_, RR, PIP, P_plateau_, or ΔP. For example, when only airway resistance is increased secondary to increased BMI, PIP is increased while P_plateau_ and ΔP which mainly reflect the restrictive components of the lung, will not necessarily change; however, the MP which reflects the energy dissipated by the mechanical ventilator and the intensity of MV onto the respiratory system over time will increase secondary to the increase in airway resistance [8].

Selecting the appropriate V_T_ is a key variable of intraoperative MV. Traditionally, with the intent of ameliorating atelectasis-induced hypoxia, anesthetists have historically ventilated patients with large V_T_s ranging between 10 and 15 ml/kg of ideal body weight. Influenced by the results of the ARDS Net trial, many anesthesiologists have strayed from this traditional practice and begun using lower V_T_ ventilation intraoperatively with the assumption that healthy patients also need ‘lung protection’ from the detrimental effects of high tidal volumes [29, 30]. Our data show that although intraoperative V_T_/IBW was higher in patients who developed PPCs (9.1 ml/kg) than in patients who did not develop PPCs (8.6 ml/kg), V_T_/IBW was not a strong indicator for the occurrence of PPCs at a threshold of 9 ml/kg. Similar to our current findings, previous studies have reported that both low [6, 31] and high intraoperative V_T_s [30, 31] were strongly associated with PPCs.

Our study indicates that PIP in patients who developed PPCs was higher than in patients who did not develop PPCs and that higher PIP (i.e., PIP ≥ 19 cmH_2_O) was not associated with increased risk of PPCs in low-risk surgical patients. This is in contrast to the Schultz et al. study that reported higher PIP to be associated with an increased risk of PPCs [30]. The differences between the current study and the Schultz et al. study are mainly that in the current study, only low-risk surgical patients were included, while Schultz et al. included both low- and high-risk patients; furthermore, the cut-off value for the PIP in the Schultz study was ≥ 20 cmH_2_O while it was ≥ 19 cmH_2_O in the current study, and although this difference might not look clinically significant, it could still be of potential importance, as the analysis suggests that for every increase of 1 cmH_2_O in peak pressure, there is a 3% increase in the odds ratio for the development of PPCs [30].

Our study also shows that PEEP was not significantly different in patients with and without PPCs. Additionally, PEEP was not found to be a significant predictor of PPCs in our patient population. In accordance with our findings, Pelosi et al. and Bluth et al. showed that strategies with high PEEP levels during open abdominal surgery do not protect against PPCs in normal and obese patients and that PEEP levels were not associated with PPCs [32, 33].

Our results show that P_plateau_ in patients who developed PPCs was not significantly different from that in patients who did not develop PPCs. However, at a threshold of 17 cmH_2_O, the P_plateau_ was found to be a significant predictor of PPCs in our patient population. This is in agreement with recently published evidence showing an association between increased P_plateau_ and the development of PPCs [34, 35]. However, our multivariable logistic regression did not confirm P_plateau_ as a strong and significant predictor of PPCs.

Our results also show that the driving pressure (ΔP), defined as P_plateau_ minus PEEP, was not significantly different in either patients who developed or those who did not develop PPCs. Furthermore, higher ΔP (≥ 13 cmH_2_O) was not found to be a strong predictor of PPCs in our patient population. Significant ΔP excursions can generate high alveolar pressures without damaging the alveoli since alveolar damage is more closely related to the strain, energy and intensity of the MV (i.e., magnitude of the repetitive cyclic stretch) than to the maximal level of stretch [8, 36].

To our knowledge, the current study is the first to assess the intensity of mechanical ventilation as reflected by the “accurate” mechanical power in the intraoperative setting and investigate its role in the development of postoperative pulmonary complications in low-risk surgical patients. However, there are still several limitations. The study is an observational, single center study that includes a relatively small number of patients. As an observational study, only associations (and not direct relationships) can be formulated. Another methodological limitation secondary to the observational nature of the study is the fact that the ranges in which relevant variables included in the determination of the mechanical power were changed during clinical routine management of our patients were low. This can limit the interpretation of the predictive values of different parameters as well as MP; a future prospective interventional study with significant changes and variations in PEEP, VT, and RR will relevantly increase the interpretation of the predictive value of intraoperative MP. In addition, the patients included were only low-risk surgical patients ventilated with a specific mode of mechanical ventilation. Also, although we did not perform a sample size calculation a priori, we conducted a post hoc power analysis based on the observed differences between the means of mechanical power of the patients with and without postoperative pulmonary complications while considering their respective standard deviations and the number of patients per each group. The power analysis indicated that for an observed difference of 1.8 J/min between means of MP, with standard deviations of 3.7 and 4.5, and with 141 and 77 patients per group, the achieved power of our study was calculated to be 84.9% which is considered a high power. Nevertheless, the current study provides valuable insights into a new and easily determined parameter, the MP, that takes into consideration not only the settings and parameter of mechanical ventilators but also the patient characteristics. Further studies should be conducted to include data from multiple centers with higher number of patients and to include high-risk surgical patients.

## Conclusions

This prospective observational cohort study shows that a substantial proportion of low-risk patients undergoing invasive mechanical ventilation are at risk for developing postoperative pulmonary complications. These patients receive higher MP than patients who do not develop PPC. At a threshold greater than 12 J/min, MP was found to be a strong predictor for the occurrence of PPCs.

## Data Availability

No datasets were generated or analysed during the current study.

## Abbreviations

ARDS: Acute respiratory distress syndrome
ARISCAT: Assess Respiratory Risk in Surgical Patients in Catalonia
ASA: American society of anesthesiologists
BMI: Body mass index
C_dyn_: Dynamic compliance
CO_2_: Carbon dioxide
ICU: Intensive care unit
I:E: Inspiratory to expiratory time ratio
MAC: Minimum alveolar concentration
MP: Mechanical power
MV: Mechanical ventilation
PACU: Post anesthesia care unit
PEEP: Positive end expiratory pressure
PIP: Peak inspiratory pressure
P_plateau_: Peak alveolar pressure
PPCs: Postoperative pulmonary complications
RR: Respiratory rate
SpO_2_: Oxygen saturation by pulse oximetry
VILI: Ventilator induced lung injury
VT: Tidal volume
ΔP: Driving pressure

## References

[1] Jin Y, Xie G, Wang H (2015). Incidence and risk factors of postoperative pulmonary complications in noncardiac Chinese patients: a multicenter observational study in university hospitals. Biomed Res Int.

[2] Fernandez-Bustamante A, Frendl G, Sprung J (2017). Postoperative pulmonary complications, early mortality, and hospital stay following noncardiothoracic surgery: a multicenter study by the perioperative research network investigators. JAMA Surg.

[3] Liu J, Meng Z, Lv R (2019). Effect of intraoperative lung-protective mechanical ventilation on pulmonary oxygenation function and postoperative pulmonary complications after laparoscopic radical gastrectomy. Braz J Med Biol Res.

[4] Miskovic A, Lumb AB (2017). Postoperative pulmonary complications. Br J Anaesth.

[5] Hemmes S, Serpa Neto A, Schultz MJ (2013). Intraoperative ventilatory strategies to prevent postoperative pulmonary complications: a meta-analysis. Curr Opin Anaesthesiol.

[6] Severgnini P, Selmo G, Lanza C (2013). Protective mechanical ventilation during general anesthesia for open abdominal surgery improves postoperative pulmonary function. Anesthesiology.

[7] Barbosa FT, Castro AA, de Sousa-Rodrigues CF. Positive end-expiratory pressure (PEEP) during anaesthesia for prevention of mortality and postoperative pulmonary complications. Cochrane Database Syst Rev. 2014;(6):CD007922. 10.1002/14651858.CD007922.pub3. 10.1002/14651858.CD007922.pub3 PMC11033874 24919591

[8] Gattinoni L, Tonetti T, Cressoni M (2016). Ventilator-related causes of lung injury: the mechanical power. Intensive Care Med.

[9] Karalapillai D, Weinberg L, Serpa Neto A (2022). Intra-operative ventilator mechanical power as a predictor of postoperative pulmonary complications in surgical patients: a secondary analysis of a randomised clinical trial. Eur J Anaesthesiol.

[10] Karalapillai D, Weinberg L, Peyton P (2020). Effect of intraoperative low tidal volume vs conventional tidal volume on postoperative pulmonary complications in patients undergoing major surgery: a randomized clinical trial. JAMA.

[11] Santer P, Wachtendorf LJ, Suleiman A (2022). Mechanical power during general anesthesia and postoperative respiratory failure: a multicenter retrospective cohort study. Anesthesiology.

[12] El-Khatib MF, Shebbo F, Beresian J (2023). Appropriate adaptation of mechanical power from the ICU to the operating room. Eur J Anaesthesiol.

[13] Mazo V, Sabaté S, Canet J (2014). Prospective external validation of a predictive score for postoperative pulmonary complications. Anesthesiology.

[14] Gupta S, Fernandes RJ, Rao JS, Dhanpal R (2020). Perioperative risk factors for pulmonary complications after non-cardiac surgery. J Anaesthesiol Clin Pharmacol.

[15] Hess DR (2014). Respiratory mechanics in mechanically ventilated patients. Respir Care.

[16] Becher T, van der Staay M, Schädler D (2019). Calculation of mechanical power for pressure-controlled ventilation. Intensive Care Med.

[17] Chiumello D, Gotti M, Guanziroli M (2020). Bedside calculation of mechanical power during volume- and pressure-controlled mechanical ventilation. Crit Care.

[18] van der Meijden S, Molenaar M, Somhorst P (2019). Calculating mechanical power for pressure-controlled ventilation. Intensive Care Med.

[19] Marini JJ, Rocco P, Gattinoni L (2020). Static and dynamic contributors to ventilator-induced lung injury in clinical practice. Pressure, energy, and power. Am J Respir Crit Care Med.

[20] Ferreyra GP, Baussano I, Squadrone V (2008). Continuous positive airway pressure for treatment of respiratory complications after abdominal surgery: a systematic review and meta-analysis. Ann Surg.

[21] Canet J, Gallart L, Gomar C, ARISCAT Group (2010). Prediction of postoperative pulmonary complications in a population-based surgical cohort. Anesthesiology.

[22] Davidovich N, DiPaolo BC, Lawrence GG (2013). Cyclic stretch-induced oxidative stress increases pulmonary alveolar epithelial permeability. Am J Respir Cell Mol Biol.

[23] Serpa Neto A, Deliberato RO, Johnson AEW (2018). PROVE Network investigators. Mechanical power of ventilation is associated with mortality in critically ill patients: an analysis of patients in two observational cohorts. Intensive Care Med.

[24] Parhar KK, Zjadewicz K, Soo A (2019). Epidemiology, mechanical power, and 3-year outcomes in acute respiratory distress syndrome patients using standardized screening. An observational cohort study. Ann Am Thorac Soc.

[25] Cressoni M, Gotti M, Chiurazzi C (2016). Mechanical power and development of ventilator-induced lung injury. Anesthesiology.

[26] Dixon AE, Peters U (2018). The effect of obesity on lung function. Expert Rev Respir Med.

[27] Peralta GP, Marcon A, Carsin AE (2020). Body mass index and weight change are associated with adult lung function trajectories: the prospective ECRHS study. Thorax.

[28] Sant’Anna M, Carvalhal RF, Oliveira FDFB (2019). Respiratory mechanics of patients with morbid obesity. J Bras Pneumol.

[29] Blum JM, Fetterman DM, Park PK, et al. A description of intraoperative ventilator management and ventilation strategies in hypoxic patients. Anesth Analg. 2010;110(6):1616–22. 10.1213/ANE.0b013e3181da82e1 20385612

[30] Schultz MJ, Hemmes SNT, Serpa Neto A, et al. The LAS VEGAS investigators. Epidemiology, practice of ventilation and outcome for patients at increased risk of postoperative pulmonary complications: LAS VEGAS - an observational study in 29 countries. Eur J Anaesthesiol. 2017;34(8):492–507. 10.1097/EJA.0000000000000646 PMC5502122 28633157

[31] Futier E, Constantin JM, Paugam-Burtz C, IMPROVE Study Group (2013). A trial of intraoperative low-tidal-volume ventilation in abdominal surgery. N Engl J Med.

[32] Pelosi P, Ravagnan I, Giurati G (1999). Positive end-expiratory pressure improves respiratory function in obese but not in normal subjects during anesthesia and paralysis. Anesthesiology.

[33] Bluth T, Serpa Neto A, Schultz MJ (2019). Effect of intraoperative high positive end-expiratory pressure (peep) with recruitment maneuvers vs low PEEP on postoperative pulmonary complications in obese patients: a randomized clinical trial. JAMA.

[34] Ladha K, Vidal Melo MF, McLean DJ (2015). Intraoperative protective mechanical ventilation and risk of postoperative respiratory complications: hospital based registry study. BMJ.

[35] Hemmes SN, Gama de Abreu M, Pelosi P (2014). High versus low positive end-expiratory pressure during general anaesthesia for open abdominal surgery (PROVHILO trial): a multicentre randomised controlled trial. Lancet.

[36] Amato MBP, Mead MO, Slutsky AS (2015). Driving pressure and survival in the acute respiratory distress syndrome. N Engl J Med.

